# Gene expression profiles of precursor cells identify compounds that reduce NRP1 surface expression in macrophages: Implication for drug repositioning for COVID-19

**DOI:** 10.3389/fcvm.2024.1438396

**Published:** 2024-10-24

**Authors:** Akira Iwata, Sarvesh Chelvanambi, Takaharu Asano, Mary Whelan, Yuto Nakamura, Elena Aikawa, Yusuke Sasaki, Masanori Aikawa

**Affiliations:** ^1^ Center for Interdisciplinary Cardiovascular Sciences, Cardiovascular Division, Department of Medicine, Brigham and Women's Hospital, Harvard Medical School, Boston, MA, United States; ^2^ Center for Excellence in Vascular Biology, Cardiovascular Division, Department of Medicine, Brigham and Women's Hospital, Harvard Medical School, Boston, MA, United States; ^3^ Channing Division of Network Medicine, Department of Medicine, Brigham and Women’s Hospital, Harvard Medical School, Boston, MA, United States

**Keywords:** COVID-19, L1000-based Connectivity Map, SARS-CoV-2, Neuropilin 1, drug screening, drug development, gene expression profiling, macrophage

## Abstract

Coronavirus disease 2019 (COVID-19) is transitioning from a pandemic to an endemic phase through recurring mutations. Initial efforts focused on developing strategies to mitigate infection of lung epithelial cells which are the primary targets of the SARS-CoV-2 virus using the affinity of the spike protein to human ACE2 receptor. SARS-CoV-2, however, infects additional cell types present in the lung such as macrophages through the alternate entry receptor Neuropilin 1 (NRP1). Developing novel therapeutic strategies to prevent SARS-CoV-2 infection of cells crucial for immunosurveillance could thus be integral to treat post-acute sequelae of COVID-19 (PASC). Since traditional drug development process takes a long time, it is imperative to establish new strategies that can be rapidly deployed to combat the dynamic nature of COVID-19 evolution and to contribute to prevention of future pandemics. We obtained the gene expression profiles of THP-1 monocytes from L1000-based Connectivity Map using CLUE, cloud- based software platform for the analysis of perturbational datasets to identify compounds that could reduce the expression level of NRP1. Out of 33,590 compounds, we analyzed the profiles of 45 compounds for their ability to reduce NRP1 expression. We selected the top five small molecule inhibitors predicted to decrease the expression of NRP1 for validation studies. All five selected compounds showed low cytotoxicity at tested doses and their ability to reduce NRP1 surface expression was evaluated in THP-1 monocytes, THP-1-derived macrophage like cells and human peripheral blood mononuclear cell (PBMC)-derived primary macrophages. Five compounds with the largest predicted reduction of NRP1 expression decreased macrophage NRP1 surface expression measured using flow cytometry and fluorescent microscopy assays in both cell line and primary macrophages. Using our computational approach, we identified 45 compounds that could potentially decrease NRP1 surface expression in macrophages based on their effect on THP-1 monocytes. Validation studies showed that such an approach can help to identify compounds for drug repositioning in target cells that are absent in the L1000 database. Our proposed approach can be applicable for the rapid compound exploration to combat novel cell types that SARS-CoV-2 targets for infection and could provide molecular bases for the development of new drugs.

## Introduction

A pandemic outbreak of severe acute respiratory syndrome coronavirus 2 (SARS-CoV-2) has caused coronavirus disease 2019 (COVID-19), which spread throughout the world. Rapid mutation of the virus gave rise to multiple variants that were circulating within the global human population and made it challenging to develop the COVID-19 treatment, despite the rapid development and approval of various mRNA vaccines and antiviral drugs, such as Nirmatrelvir and Ensitrelvir. In 2024, COVID-19 has turned into an endemic phase. To develop the therapeutics remedy, the initial research efforts were focused on identifying the host cell receptors, such as angiotensin-converting enzyme 2 (ACE2) from lung cells, to develop inhibitors (1). As COVID-19 becomes an endemic disease, it has become increasingly important to redirect efforts to prevent overactive immune inflammatory response, caused by inflammatory macrophages. SARS-CoV2 induced macrophage activation, reduced immune surveillance, and an impaired ability to clear the SARS-CoV2 infection. Recent studies showed that SARS-CoV2 uses an alternate entry receptor, Neuropilin 1 (NRP1) to infect cells not expressing ACE2 (2–4). NRP1 was found to be highly expressed in macrophages within atherosclerotic lesions, particularly in plaque-associated foamy macrophages (5). Experimental silencing of NRP1 gene reduced SARS-CoV2 infectivity of human primary macrophages (5). Overall, NRP1 is involved in mediating SARS-CoV-2 infection of the atherosclerotic plaque, indicating its importance in viral pathogenesis. Our previous reports also suggest that expression of NRP1 could be associated with increased acute inflammatory responses in coronavirus infection (6). Similarly, NRP1 has been implicated in the infection of macrophage reservoirs in the nasal epithelium (3) as well as astrocytes in the brain (7). This creates the need for adaptable drug discovery pipelines to target specific reservoirs of SARS-CoV2 infection to treat the diverse range of outcomes seen in COVID-19 patients such as neurocognitive disorders and cardiovascular complications.

The process of drug development involves several steps, including compound discovery, screening, and clinical trials. Developing hit compounds from screening into the lead candidate compounds and conducting clinical trials may take more than 10 years, often due to unexpected off-target effects resulting in adverse events. To address this issue, drug repositioning approach has been gaining increased attention. This approach contributes to discovering new indications for existing drugs, by saving time and cost compared to traditional drug screening (8, 9). The main reason is that existing candidate compounds have already proven their safety in preclinical and clinical trials. Moreover, they are cost-effective due to their established synthesis methods. In addition, computational drug repositioning has been evolved in recent years (10). This approach requires the publicly available large-scale biological data for existing drugs. Broad Institute constructed a database of gene expression profiles induced by exposing a variety of cell types to various perturbations including small molecules, called the Connectivity MAP (CMap). The expansion of this database has resulted in more than a million gene expression profiles using more than 30,000 small molecules and 200 cell types through the introduction of L1000 assay technology (11, 12). L1000-based Connectivity Map is one of the largest libraries of integrated network-based cellular signatures available and many studies have been conducted using this database. Candidate molecules could be predicted by comparing L1000 perturbation profiles and the disease-specific profiles obtained by the gene expression omnibus (GEO). An important application of L1000-based CMap is rapid drug repositioning, which is based on finding small molecules that cancel or mimic differences in gene expression caused by diseases (13, 14). In addition, these drug repositioning approaches using L1000-based CMap can save time and cost during the drug development process.

Recently, we reported the *in-silico* drug screening approach using L1000-Based Connectivity Map and its application to the entry receptor for the SARS-CoV-2 virus, ACE2 (15) and another disease context, including atherosclerosis (16). Using this method, we could rapidly identify small molecule inhibitors of target gene in specific cell lines. While the L1000 database contains 248 types of cells, only 12 of those are primary or non-tumor cells. Of note, there are no non-tumorous immune cell types in this database currently. We used THP-1 monocytes, a tumor-derived monocytic cell line, which is a precursor to our cell type of interest, macrophages.

In this study, aiming at further utilizing our rapid compound exploration method, we computationally identified small molecule compounds that were predicted to decrease NRP1 expression using the gene expression profiles for THP-1 monocytes as an alternative to expression profiles for macrophages. Furthermore, we conducted validation experiments in both THP-1 derived macrophage-like cells and peripheral blood mononuclear cell (PBMC)-derived macrophages to verify the effect of the compounds predicted to decrease NRP1 surface expression using flow cytometry and fluorescence microscopy. Overall, this study suggests the potential to rapidly identify compounds that decrease NRP1 expression in macrophages, which is not covered by L1000, using gene expression levels of precursor THP-1 monocytes.

## Materials and methods

### Gene expression profiles in L1000-based CMap dataset

The level 5 gene expression profiles of L1000-based CMap were downloaded from clue.io. This Level 5 dataset contains over 10,000 genes with differential expression values, and 1,201,944 profiles were induced by several conditions. This dataset consists of 46934 perturbagens on 248 types of cells, 162 doses, and 23-time points. The profile values represent the level of mRNA expression in comparison to a control (the background of the plate). In each gene expression profile, 12,328 genes are measured directly (called landmark genes). 9,196 genes are well inferred, and their expression level did correlate with the actual measured levels with p values ≤0.05, and the remaining 2,154 genes are less-well inferred genes. The well-inferred Genes include both NRP1 and ACE2.

### Cell culture and reagents

THP-1 monocytes (TIB-202, ATCC) were cultured in RPMI1640 (MT10040CV, Gibco) supplemented with 10% fetal bovine serum (Gibco) and 1% penicillin-streptomycin (15140163, Gibco). THP-1 cells were differentiated into macrophage-like cells using 10 ng/ml phorbol 12-myristate 13-acetate (PMA) (Catalog #P8139, Sigma) for 48 h followed by 24 h rest before experiments. Human primary peripheral blood mononuclear cells were isolated from buffy coat using the Ficoll-based density gradient centrifugation method. Monocytes were allowed to adhere to the plate for 1 h and non-adhered leucocytes were removed. Monocytes were differentiated into macrophages by culturing cells with RPMI + 5% human serum for 7–10 days (17–27). In total, cells obtained from 8 different de-identified healthy donors were used in this study. ONO-4059 (Catalog # 32957), gefitinib (Catalog # 13166), bosutinib (Catalog # 12030), doxorubicin (Catalog #15007), and docetaxel (Catalog #11637) were purchased from Cayman Chemicals and resuspended in DMSO according to manufacturer instructions and used at indicated doses in the study. Cells were treated for 12 h using all compounds prior to evaluation of viability using Propidium Iodide (Catalog#R37108, Thermofisher) and surface expression of NRP1.

### Flow cytometry

Cells were stained for flow cytometry using viability dye (Propidium iodide) and either isotype (Catalog #407107, Biolegend) or anti-NRP1 antibody (Clone 12C2, Catalog #354503, Biolegend) for 30 min on ice. Cells were washed 3× using PBS followed by fixation with 4% paraformaldehyde fixation. Surface expression of NRP1 was measured using Cytek Aurora flow cytometer (Cytek Bioscience). FCS files were analyzed in FlowJo version 10.10.0.

### Immuno-fluorescent staining with high content imaging (HCI) for quantifying NRP1 expression

Cells were fixed using 4% paraformaldehyde for 15 min at room temperature and washed 3 times with PBS. Cells were then stained overnight with either isotype (Catalog #407107, Biolegend) or anti-NRP1 antibody (Clone 12C2 Catalog #354503, Biolegend) with gentle agitation. Cells were washed 3 times using PBS followed by counterstain for nucleus (DAPI) and actin cytoskeleton (Phalloidin) expression of NRP1 was measured using Molecular Devices ImageXpress Pico (Molecular Devices). Cell counting was performed using DAPI staining and cellular NRP1 expression was quantified using 3-cell scoring program in CellReporter Xpress (Molecular Devices). Mean fluorescence per cell, total NRP1 fluorescence and percent of NRP1+ cells were calculated using this program.

### Statistical methods

All data was analyzed using Graphpad Prism v10 (GraphPad Software). For multiple group comparisons in THP1 monocytes and THP1 derived macrophages, One-way ANOVA followed by post-hoc multi-group comparisons were performed with *p* < 0.05 used to determine statistical significance. For comparison across donors in primary cells, values for NRP1 expression were averaged across technical replicates and fold change to vehicle treated macrophages (control) was calculated for each treatment. One-sample *t*-test was used to compare the value across multiple donors to value of 1.0 (normalized to control) with *p*-value < 0.05 used to determine compounds that reduced NRP1 surface expression with statistical significance.

## Results

**Figure 1:**
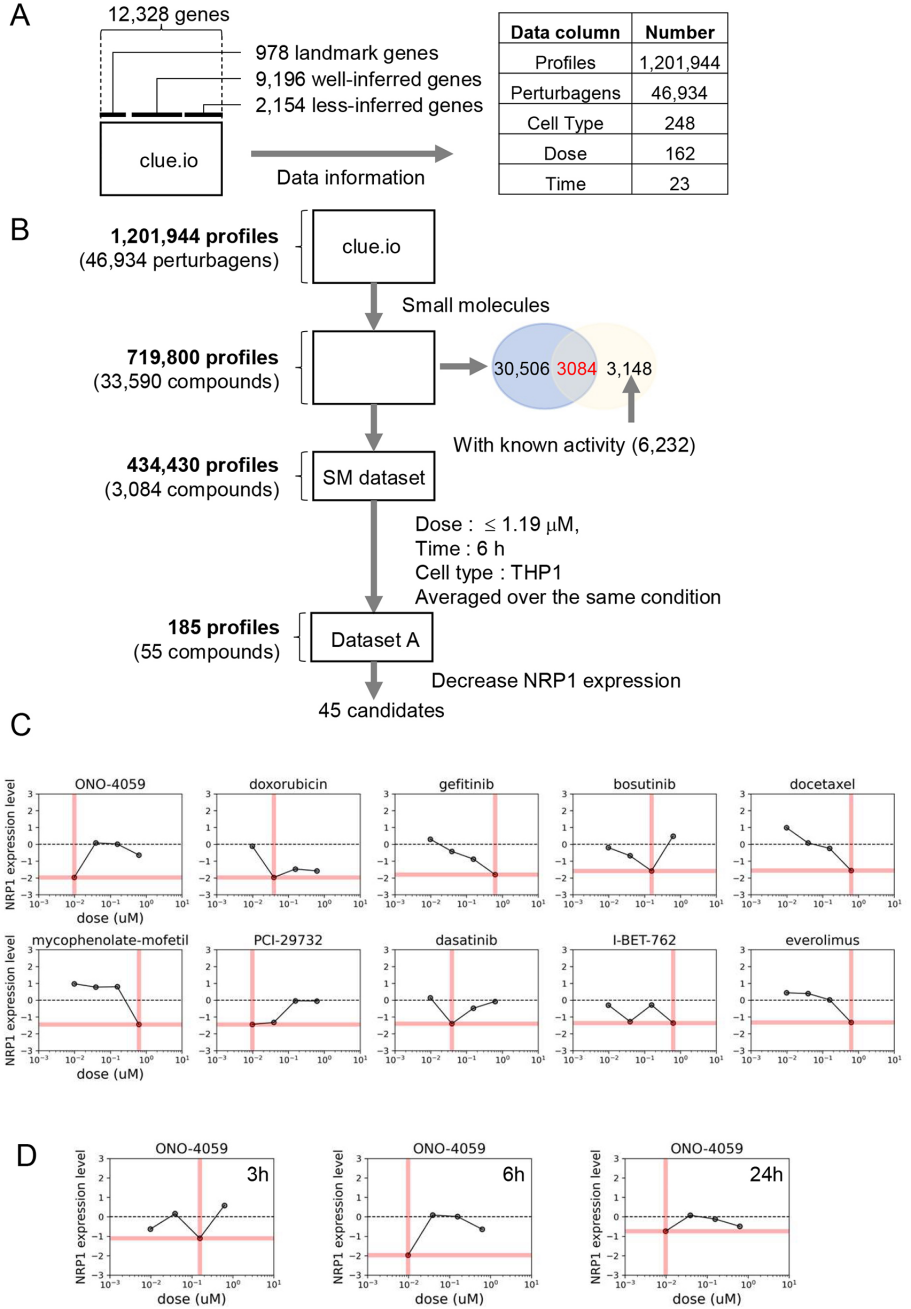
Drug screening process of reducing NRP1 expression in THP-1 monocytes. **(A)** The Structure of L1000-based connectivity MAP (CMap) dataset. **(B)**) Drug Identification process (workflow). **(C)** Dose-response relationships of NRP1 expression in top 10 small molecules in THP-1 monocytes at 6 h time point. Pink bars indicate the dosages in each small molecule summarized in Table 1. **(D)** Dose-response relationships of NRP1 expression in ONO-4059 in THP-1 at 3, 6 and 24 h time points.

The goal of this study was to identify small molecules that decrease NRP1 expression in macrophages. As the L1000-based CMap dataset contains many profiles under multiple conditions, including perturbagens, cell types, doses, and time points, appropriate filters are necessary to narrow it down (Figure 1A). To find the desired molecules, we applied the following filters to this dataset according to the indicated filters (Figure 1B).

### Selection of small molecules from L1000-based CMap dataset with drug repurposing hub

Perturbagens include reagents such as small molecules, CRISPR/Cas9 and short hairpin RNA. Drug Repurposing Hub is an open-access repository of 6,232 molecules, which is curated and annotated of FDA-approved drugs, drugs in clinical trials, and pre-clinical tool compounds with resource information. In addition, it describes the mechanisms of action of these compounds (drug information version: 3/24/2020) (28). Among the 33,590 molecules contained in L1000-based CMap dataset, 3,084 small molecules overlap with 6,232 drugs in this repository. We then extracted the 434,430 gene expression profiles with conditions treated with these 3,084 small molecules.

### Dosage, time, and cell type selection

Each profile was measured at various dosages, time points, and cell types in the L1000-based CMap dataset. To facilitate comparison and easy handling of data, several doses from a similar range were converted to single values in Supplementary Table S1. Among dosages, we eliminated high dosages both from concerns about toxicity and drug discovery and we applied the filter of 1.19 µM (Figure 1B). Next, in the narrowed-down profiles by dose selection, the profiles with the most data continue in the order of 24 h, 6 h, and 3 h. In terms of drug discovery, the profiles measured over 24 h were excluded to identify fast-acting drugs. For the subsequent analysis, we focused on a larger amount of data from 6 h timepoint to compare between compounds. Despite applying the same small molecules to each type of cell, the gene expression profiles differ, so that gene expression profiles should be used in a specific type of cell. Ideally, we would obtain profiles measured in macrophage-derived cells from the L1000-based CMap dataset. However, in the absence of data for macrophages, we chose the profiles measured in THP-1 monocytes since these are routinely used to evaluate inflammatory responses in cardiovascular disease and contained the most number of compounds and timepoints tested in the L1000-based CMap. Finally, after filtering, 190 profiles were obtained and we averaged profiles collected multiple times, resulting in the final set of 185 profiles treated by 55 small molecules in THP-1 for 6 h.

### Identification of small molecules that decrease NRP1 expression in THP-1

We focused on the expression level of NRP1 in the preprocessed dataset, including both compounds that could increase and decrease NRP1 expression. Among 55 compounds, we selected 45 small molecule inhibitors that were predicted to decrease NRP1 expression. We then sought to extract the combinations of 45 small molecules and their doses that most significantly decrease the expression of NRP1 in the preprocessed dataset. Among these combinations, the top 10 small molecules that most significantly decrease the expression level of NRP1 are shown in Table 1. Furthermore, the preprocessed dataset contained NRP1 expression levels at 4 dose points, and Figure 1C depicts the dose-response relationship of NRP1 expression in each small molecule. A dose-dependent decrease in NRP1 expression tended to be observed in several small molecules in the ranges up to their optimal doses. In the result, we expected these small molecules to decrease NRP1 expression at the appropriate dose in THP-1. We then observed the dose-response relationship of ONO-4059 at each time point (i.e., 3 h, 6 h, and 24 h) (Figure 1D). Among three time points, this molecule was predicted to reduce NRP1 expression at 6 h. As a result of these findings, we selected the top combinations of 5 small molecules and their doses that could decrease the expression of NRP1 at 6 h for subsequent validation studies.

**Table 1:** The top 10 small molecules identified, NRP1 expression levels, dose and known activity.

Rank	Compound name	Expression level	Dose	Known activity
1	ONO-4059	−1.972	0.01 μM	Bruton’s tyrosine kinase (BTK) inhibitor
2	Doxorubicin	−1.968	0.04 μM	Topoisomerase inhibitor
3	Gefitinib	−1.797	0.66 μM	EGFR inhibitor
4	Bosutinib	−1.576	0.125 μM	Abl kinase inhibitor|Bcr-Abl kinase inhibitor|SRC inhibitor
5	Docetaxel	−1.563	0.66 μM	Tubulin polymerization inhibitor
6	Mycophenolate-mofetil	−1.443	0.66 μM	Dehydrogenase inhibitor|inositol monophosphatase inhibitor
7	PCI-29732	−1.438	0.01 μM	Bruton’s tyrosine kinase (BTK) inhibitor
8	Dasatinib	−1.396	0.04 μM	Bcr-Abl kinase inhibitor|ephrin inhibitor|KIT inhibitor|
				PDGFR tyrosine kinase receptor inhibitor|SRC inhibitor|
				Tyrosine kinase inhibitor
9	I-BET-762	−1.351	0.66 μM	Bromodomain inhibitor
10	Everolimus	−1.324	0.66 μM	mTOR inhibitor

### Regulation of surface expression of NRP1 in THP-1 monocytes

**Figure 2:**
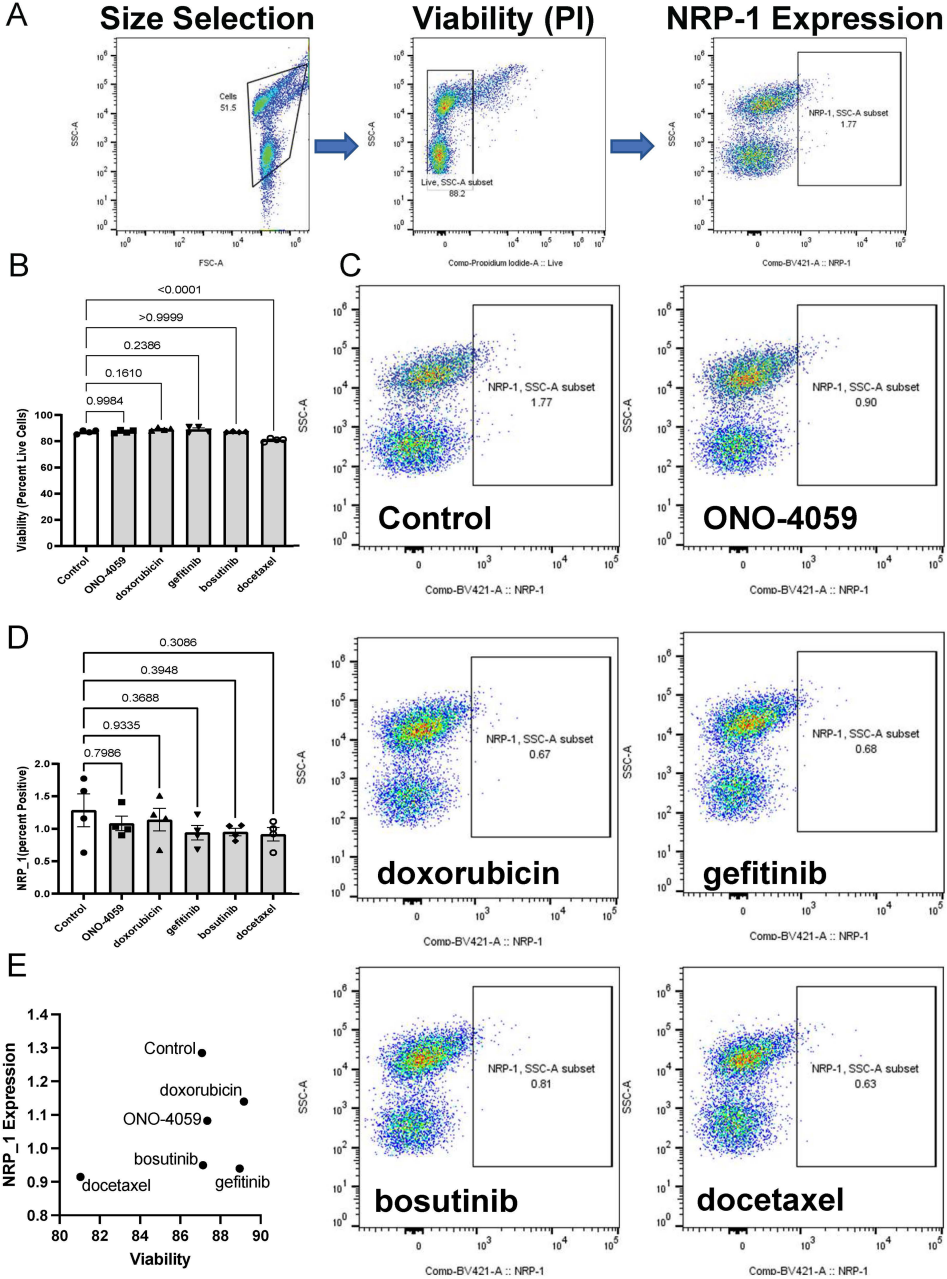
Cell surface NRP1 expression analysis in THP-1 monocytes. **(A)** Gating strategy for the fluorescent activated cell sorter (FACS). **(B)** Cell viability after treating the top 5 candidate small molecules (ONO-4059, doxorubicin, gefitinib, bosutinib, and docetaxel). **(C)** Cell surface NRP1 expression analysis after treating the top 5 candidate small molecules. **(D)** Summary of cell surface NRP1 expression. **(E)** The correlation between NRP1 expression and cell viability. Data were shown as mean (bar) plus individual value (dots), *n* = 4 per group. One-way ANOVA followed by post-hoc multi-group comparison test was used to assess statistical differences.

Our analysis of L1000 CMap identified multiple compounds predicted to decrease NRP1 expression in THP-1 monocytes. We selected the top 5 compounds based on their predicted ability to decrease NRP1 expression. Since NRP1 is a surface receptor that acts as an entry receptor for SARS-CoV2, we chose to measure the surface protein expression of NRP1 as the ideal parameter to quantify the ability of these predicted compounds to decrease NRP1 expression and thereby potentially prevent SARS-CoV2 infection. We first screened whether these compounds would impact cell viability through flow cytometry (Figure 2A) using propidium iodide staining. We observed that all compounds had minimal effect on THP-1 monocyte viability (Figure 2B) at the dosage predicted by our L1000 CMap analysis. We next investigated the capacity of these compounds to reduce surface expression of NRP1 (Figure 2C) and observed that these compounds only had marginal effects on reducing NRP1 expression in THP-1 monocytes (Figure 2D). However, baseline expression of NRP1 at the surface of THP-1 monocytes itself was considerably low. Finally, we compared the ability of these compounds to reduce NRP1 surface expression with minimal effect on cellular viability (Figure 2E).

### Regulation of NRP1 surface expression in THP-1 derived macrophage-like cells

**Figure 3:**
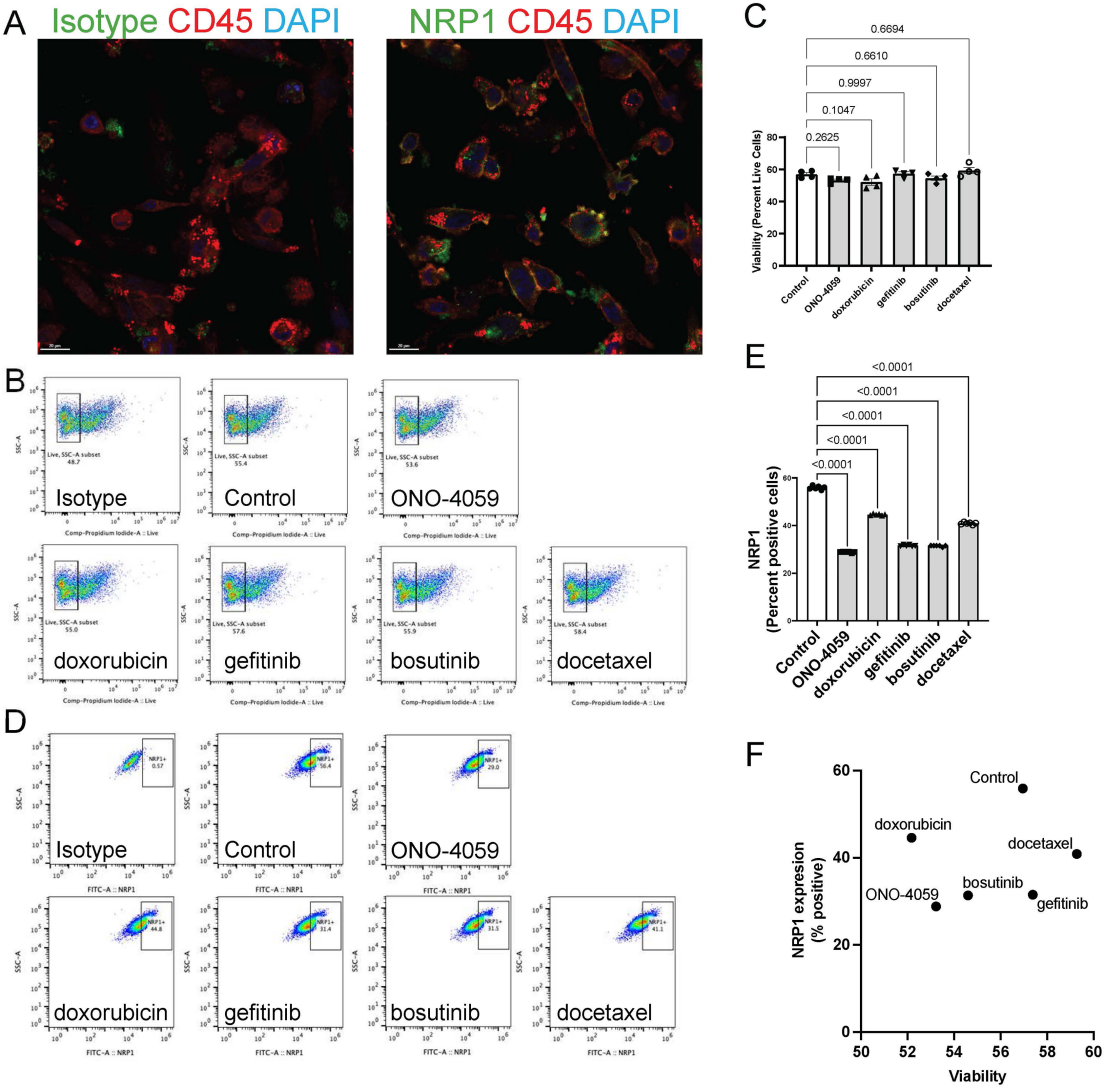
Cell surface NRP1 expression analysis in THP-1 derived macrophages. **(A)** The immunocytochemistry staining on cell surface NRP1 expression in THP-1-derived macrophage-like cells (Green: NRP1 or isotype, Red: CD45, and Blue: DAPI). **(B,C)** Live cell quantification analysis after treating the top 5 candidate small molecules. Representative of cell viability after treating the top 5 candidate small molecules. Scale bar, 20 μm. **(B)** (ONO-4059, doxorubicin, gefitinib, bosutinib, and docetaxel) and summary of cell viability **(C–E)** Cell surface NRP1 expression analysis after treating the top 5 candidate small molecules. Representative images **(D)** and summary of cell surface NRP1 expression **(E,F)** The correlation between NRP1 expression and cell viability. Data were shown as mean (bar) plus individual value (dots), *n* = 4 **(C)** and 6 **(E)** per group. One-way ANOVA followed by post-hoc multi-group comparison test was used to assess statistical differences.

To investigate the appropriate cell type in which NRP1 surface expression is linked to SARS-CoV2 infection, we evaluated whether THP-1 cell differentiated into macrophage-like cells using PMA would induce NRP1 surface expression. Indeed, we observed surface expression of NRP1 in THP-1-derived macrophage-like cells using confocal microscopy (Figure 3A). We also validated these findings using flow cytometry in cells which were not permeabilized to show that macrophages would be an optimal candidate to validate the ability of these compounds to reduce surface expression of NRP1. We observed that none of the compounds had a significant impact on cell viability (Figures 3B,C) suggesting these compounds have minimal cytotoxicity in THP-1 derived macrophage-like cells. Flow cytometry analysis was performed to describe the ability of these compounds to reduce surface expression of NRP1 in THP-1 derived macrophage-like cells (Figure 3D). We observed that all five compounds tested caused a statistically significant reduction in the percentage of NRP1-positive cells (Figure 3E). We compared the ability of these compounds to reduce NRP1 surface expression with its effect on cellular viability (Figure 3F) to identify compounds with the best safety profile for macrophage cytotoxicity and maximal NRP1 downregulation.

### Regulation of NRP1 surface expression in human primary PBMC-derived macrophages

**Figure 4:**
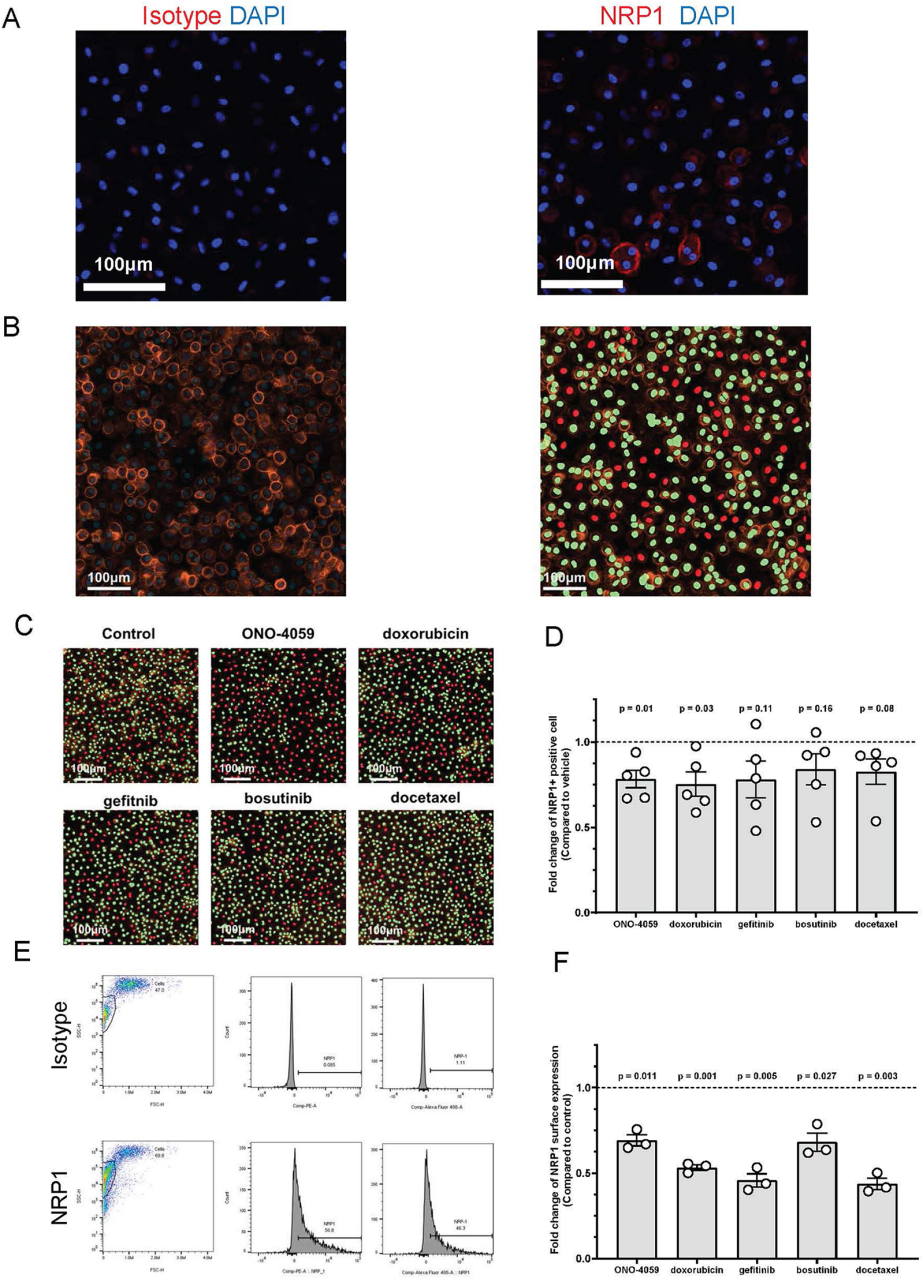
Cell surface NRP1 expression analysis in human primary peripheral blood mononuclear cell (PBMC)-derived macrophages. **(A)** The immunocytochemistry staining on cell surface NRP1 expression in PBMC-derived macrophages (Red: NRP1 or isotype, and Blue: DAPI). Scale bar, 100 μm. **(B)** Cell surface NRP1 surface expression (Orange) analysis in high-content imaging using ImageXpress Pico. Cell masks created by cell scoring program in CellReporter Xpress for cells with positive NRP1 expression (green) and cells with negative NRP1 expression (red). **(C)** Cell masks for NRP1 positive (green) and negative (red) cells after treating the top 5 candidate small molecules (ONO-4059, doxorubicin, gefitinib, bosutinib, and docetaxel). **(D)** Summarized results for five independent de-identified healthy donors with each dot representing each donor. **(E)** Gating strategy for the fluorescent activated cell sorter (FACS). **(F)** Cell surface NRP1 expression analysis after treating the top 5 candidate small molecules in FACS. Summarized with NRP1 MFI ratio corrected by donor. Summarized results for three independent de-identified healthy donors with each dot representing each donor. For figures **(D,F)**, the average NRP1 expression was normalized to control (vehicle treated) sample in each donor. One-sample *t*-test was used to compare mean NRP1 expression across donors. *P*-value < 0.05 was used to determine statistically significant changes.

SARS-CoV2 infection of innate immune cells such as macrophages has been shown to be related using the NRP1 receptor *in vivo*. Therefore, we isolated human primary PBMCs from de-identified healthy donors and differentiated them into macrophages. NRP1 surface expression was found to be heterogeneous and present within a subset of macrophages through confocal microscopy (Figure 4A). Using high content imaging, we created masks for NRP1 positive (green) and NRP1 negative (red) cells (Figure 4B). We added all five compounds at the doses predicted by our L1000 CMap analysis and measured surface expression of NRP1 using high-content imaging (Figure 4C). Across five independent donors, we observed that our top 2 predicted compounds, ONO-4059 and doxorubicin were successfully able to reduce the percentage of NRP1-positive cells with statistical significance using high content imaging (Figure 4D). To sample a larger number of cells per condition and more accurately measure mean NRP1 surface expression at single cell resolution, we supplemented our high content imaging approach with flow cytometry (Figure 4E). Here we observed that in three different, de-identified healthy donors, all five predicted compounds were able to reduce the mean fluorescence intensity of NRP1 surface expression compared to vehicle treated control primary PBMC-derived macrophages (Figure 4F). Our findings using high-content imaging (Figure 4D) and flow cytometry (Figure 4F) suggest that our predicted compounds using L1000-based CMap dataset were validated in their capacity to regulate NRP1 surface expression in human primary PBMC-derived macrophages across multiple donors.

## Discussion

Based on the drug repositioning approaches for the L1000-based CMap dataset, we identified 45 candidate small molecules that could potentially decrease NRP1 surface expression in macrophages. The gene expression profiles induced by exposing macrophages were not included in this dataset. We, therefore, employed alternative approaches to identify candidate small molecules using THP-1 monocytes, which are routinely used as a macrophage-like cell after differentiation using PMA. We expected these candidates with the ability to decrease NRP1 mRNA to reduce NRP1 surface expression in macrophages. We verified the effect of the candidate compounds through validation experiments in both THP-1-derived macrophages and PBMC-derived macrophages using flow cytometry and fluorescence microscopy. Our study successfully translates predictions made in tumor cell lines such as THP1 monocytes to primary non-cancer cells (PBMC-derived macrophages from 8 different de-identified healthy donors) showing the generalizability of the predictions made using L1000.

We previously identified small molecules that could potentially decrease ACE2 mRNA levels in the lung epithelial cell lines by processing the L1000-based CMap dataset (15). Most candidates were shown to decrease ACE2 surface expression in the target cells BEAS-2B derived from the same organ. This current study revealed the potential to identify compounds that decrease gene expression in macrophages using the gene expression level of precursor THP-1 monocytes. These results indicate that our proposed approach could be adapted to identify compounds for drug repositioning in several cells using the precursor cells. Since primary cell data are limited in the L1000-based CMap dataset, this approach would make it possible to identify the compounds that modulate target gene expression using precursor cells, which is often used in primary cell studies. We have also employed such approaches to validate candidate small molecules using *in vivo* studies reversing disease phenotype (16, 26). Future studies can extend our findings into *in vivo* studies to accelerate translation to preclinical testing.

However, there are a few restrictions on using the L1000-based CMap dataset. First, as mentioned in the method, many genes are not covered in the L1000 dataset, resulting in the limitation of drug discovery for half of the human protein-coding genes. To address this limitation, a deep-learning model that transforms L1000 profiles to RNA-seq-like profiles has already been developed (29). Second, most data were obtained by performing anticancer drugs in cancer cell lines. The goal of drug repositioning is to obtain the candidate drugs without inducing high toxicity. It is noteworthy that in this study we extracted the small molecules that decrease NRP1 surface expression using a dose filter and identified the candidate compounds without significant cytotoxicity or cell viability reduction in each assay. One advantage of our approach is that the L1000-based CMap dataset contains data from multiple cell types. While we focused on monocytes and monocyte-derived macrophages in this study, the effect of drug treatment on regulation of NRP1 expression in other cell types can also be obtained. For example, the L1000 contains 12 different cell types treated with ONO-4059. Future studies investigating the potential reduction of NRP1 in other cell types and tissues can also be guided using these approaches. On the other hand, since the L1000 is built on data measured primarily from cell lines, it is difficult to make inferences about the sex-dependent and age-dependent effects of drug compounds. In our study, we used the same cell line (THP-1) as the L1000 and supplemented these findings in macrophages differentiated from PBMC of de-identified healthy donors. Future studies interested in investigating sex-dependent and age-dependent effects of such compounds in regulating NRP1 expression can use appropriately powered validation models (e.g., male and female mice) to answer these questions.

In recent years, drug development has become more challenging due to the depletion of drug targets. However, there is still significant demand for treatments that slow, stop or reverse disease progression. L1000-based CMap database has been used for drug repositioning to identify small molecules that induce gene expression profiles similar to or opposite to those caused by diseases (26, 30). Increasing publicly available large-scale biological data would facilitate computational drug repositioning. If the profiles induced by exposing various cells using drugs already proven safe are expanded, we expect that drug discovery using L1000-based drug repositioning would aid in the expansion of new indications for existing drugs.

During the evolution of viral epidemics, viruses such as SARS-CoV-2 rapidly acquire mutations on their surface receptors, which impacts the tropism of these viruses. As a result, SARS-CoV2 could continue to infect multiple cell types using diverse range of entry receptors and co-receptors. While traditional vaccination and antiviral drug strategies that prevent viral replication are key tools in curbing the spread of viral infection, our approach provides a strong complementary strategy. Electronic medical record-based approaches have been used to identify at-risk populations, such as obese individuals who are at increased risk for productive infection upon exposure to SARS-CoV2 virus. Our approach to finding drugs that could be repurposed to prevent the spread of infection could be a powerful tool to mitigating spread of viral infection (31). Rapidly screening compounds to identify specific candidates to prevent infectivity of cells such as macrophages, endothelial cells or neurons could serve as a supportive strategy to prevent the diverse range of post-acute sequelae of COVID-19. Specifically, therapies targeting cell types which play a key role in inflammation and resolution of inflammation could be a potent strategy to reduce the risk for post-acute sequelae of COVID-19 (32). While this approach facilitates rapid identification of compounds that could reduce surface expression of NRP1, the mechanisms through which these compounds are able to induce these effects are unknown. This is a limitation which requires orthogonal studies to investigate how these compounds are able to regulate NRP1 expression. For certain patients with pre-existing atherosclerosis, inhibition of NRP suppresses infection but cause an aberrant pro-inflammatory response. NRP1-null macropjages are known to increase the release of inflammatory cytokines, such as IL-6. However, the inclusion of parameters to test cell viability as utilized in this study can help further filter compounds with best safety profile and maximal efficacy. Furthermore, the use of compounds which have already obtained FDA approval and have well documented safety profile could be an additional layer in the selection of compounds to combat the infection of SARS-CoV2 into diverse cell types. In our proposed model, drugs with prior FDA approval will be repurposed for transient reduction of NRP1 expression in macrophages to reduce the risk of infection with SARS-CoV2. While some studies have shown that myeloid specific knockout of NRP1 could increase pro-inflammatory responses (33, 34), our approach is aimed at short-term, transient downregulation of NRP1.

Many small molecules with potential anti-COVID-19 effects have been developed as our understanding of SARS-CoV2 structure and function has evolved (35). The development of new compounds and antibodies targeting NRP1 can be effective (36, 37), but a significant amount of time is required to evaluate safety of these new compounds in preclinical testing and clinical trials. In addition, rapid and large-scale supplies are essential in the event of a pandemic. The drug repositioning approach used in this study applies compounds with established synthetic methods and safety profiles. Such an approach could serve to support therapies directly targeting NRP1 as a cheaper and faster complementary therapeutic option. Future studies can compare the safety and efficacy of agents directly targeting NRP1 with our proposed drugs indirectly lowering NRP1 to help select appropriate therapeutic strategy for patients with COVID-19.

The ability of innate immune cells such as macrophages to sense and respond to viruses is regulated through a wide range of mechanisms. Poly (ADP-ribose) polymerase form a key role in sensing viruses using their macrodomain (38–40). PARPs have been shown to play a key role in the regulation of macrophage heterogeneity in response to virus infection-related factors such as interferon γ (21). Similarly, infection-associated signaling factors such as extracellular vesicles (41) and TLR signaling (42) play a role in severity of inflammatory responses. Specifically, the role of extracellular vesicle-associated viral proteins in determining chronic inflammatory responses has also been shown in HIV (43, 44), influenza (45) and SARS-CoV-2 (46). Use of L1000-based CMap approaches could be employed to find novel receptors and regulators of inflammation. While the L1000-based CMap contains interferon *γ* as a ligand in 84 conditions, it has not been evaluated in THP-1 monocytes. Therefore, we focused on unstimulated monocytes and macrophages to validate the ability of the predicted compounds to reduce NRP1 surface expression. Future studies can focus on evaluating NRP1 expression under pathophysiological stimulation such as interferon *γ* in appropriate cell types. L1000-based CMap could also be used to guide studies to reverse gene expression profiles based on disease specific stimulation. We have previously used such an approach to identify compounds to reduce the pro-inflammatory phenotype induced by interferon *γ* in human primary macrophages (26). Similar studies can be performed to repurpose drug compounds to target specific phenotypes of polarized macrophages. These could be targeted using our drug repurposing approach to reduce virus-induced chronic inflammation.

## Data Availability

The authors confirm that the data supporting the findings of this study are available within the article and its supplementary materials.
