# Supplementary material for: Gene expression profiles of precursor cells identify compounds that reduce NRP1 surface expression in macrophages: Implication for drug repositioning for COVID-19
Source: Front Cardiovasc Med. 2024 Oct 24;11:1438396. doi: 10.3389/fcvm.2024.1438396 (PMC11541348; doi:10.3389/fcvm.2024.1438396)
Supplement: Supplementary file 1 [file Table1.pdf]

1 **Supplementary TABLE S1** | Look-up table to convert doses to the representative values.

Representative Value (μM)	Range_min (μM)	Range_max (μM)
0.0001 uM	0.0000412	0.00025
0.0005 uM	0.0003	0.000749999
0.001 uM	0.00079167	0.0013333
0.002 uM	0.0015	0.00244141
0.003 uM	0.0025	0.00333333
0.004 uM	0.0035355	0.005
0.006 uM	0.005	0.00769803
0.01 uM	0.008	0.015
0.02 uM	0.0152	0.021213
0.04 uM	0.015625	0.0444127
0.025 uM	0.0230941	0.0273041
0.03 uM	0.0279576	0.0333581
0.05 uM	0.0451768	0.0540825
0.06 uM	0.055	0.0632324
0.08 uM	0.0625	0.0999996
0.07 uM	0.0692823	0.0741
0.12 uM	0.1	0.125
0.125 uM	0.122605	0.162247
0.16 uM	0.156092	0.15625
0.2 uM	0.166667	0.207847
0.22 uM	0.213856	0.230769
0.25 uM	0.24	0.27478
0.24 uM	0.25	0.25
0.3 uM	0.28284	0.333333
0.37 uM	0.336692	0.5
0.5 uM	0.44194	0.58
0.66 uM	0.5815	0.684161
0.63 uM	0.62437	0.625
0.74 uM	0.715553	0.922787
1.11 uM	0.944017	1.18
1.25 uM	1.18007	1.4453
1.67 uM	1.46023	1.94
2.22 uM	1.98	2.35479
2 uM	2	2
2.5 uM	2.3699	2.84591
3.33 uM	2.92031	4
4 uM	3.66621	5.32883
6.66 uM	5.33	7.32509
8 uM	7.33243	8.99761
10 uM	9	11
12 uM	11.0009	12.2469
12.5 uM	12.2509	13.7345
15 uM	13.7609	15000

20 uM	17.5585	22.4919
25 uM	22.6456	27.3117
30 uM	28.39	30000
40 uM	35.0021	44.4
50 uM	48	53.124
60 uM	56.78	64
70 uM	69.83	70.07
75 uM	75	75
80 uM	79.44	80
90 uM	88.8	94.8084
100 uM	95.4319	111.11
125 uM	114.269	125
150 uM	150	160
200 uM	177.6	200
250 uM	250	250
320 uM	313	316.2
625 uM	625	800
1 uM	937.5	937.5
1000 uM	1000	1000
1250 uM	1234.57	1250
1.88 uM	1875	1875
2500 uM	2500	2500
3200 uM	3162	3162.3
3700 uM	3703.7	3703.7
3.75 uM	3750	3750
5000 uM	5000	5000
7.5 uM	7500	7500
10000 uM	10000	11111.1
32000 uM	31623	33333.3
100000 uM	100000	100000
200000 uM	200000	200000
320000 uM	316228	316228
